# Common molecular mechanism of the hepatic lesion and the cardiac parasympathetic regulation in chronic hepatitis C infection: a critical role for the muscarinic receptor type 3

**DOI:** 10.1186/s12859-016-0988-7

**Published:** 2016-03-22

**Authors:** Sanja Glišić, David P. Cavanaugh, Krishnan K. Chittur, Milan Sencanski, Vladimir Perovic, Tijana Bojić

**Affiliations:** Institute of Nuclear Sciences Vinča, University of Belgrade, Center for Multidisciplinary Research, PO Box 522, Belgrade, Serbia; Benchmark Electronics, Bradford Dr., Huntsville, AL 35805 USA; Chemical and Materials Engineering, University of Alabama Huntsville, Huntsville, AL 35899 USA; Institute of Nuclear Sciences Vinča, University of Belgrade, Laboratory of Radiobiology and Molecular Genetics, PO Box 522, 11000 Belgrade, Serbia

**Keywords:** Informational Spectrum Method, Hepatitis C, Muscarinic receptor type 3, Cross-reactivity, Autonomic nervous system, EIIP, Hydrophobicity

## Abstract

**Background:**

The pathophysiological overlapping between Sjorgen’s Syndrome (SS) and HCV, presence of anti- muscarinic receptor type 3 (M3R) antibodies in SS, the role that M3R plays in the regulation of the heart rate, has led to the assumption that cardiovagal dysfunction in HCV patients is caused by anti-M3R antibodies elicited by HCV proteins or by their direct interaction with M3R.

**Results:**

To identify HCV protein which possibly is crossreactive with M3R or which binds to this receptor, we performed the Informational Spectrum Method (ISM) analysis of the HCV proteome. This analysis revealed that NS5A protein represents the most probable interactor of M3R or that this viral protein could elicit antibodies which modulate function of this receptor. Further detailed structure/function analysis of NS5A and M3R performed by the ISM method extended with other Digital Signal processing (DSP) approaches revealed domains of these proteins which participate in their crossreactivity or in their direct interaction, representing promising diagnostic and therapeutic targets.

**Conclusions:**

Application of the ISM with other compatible bioinformatics methods offers new perspectives for identifying diagnostic and therapeutic targets for complicated forms of HCV and other viral infections. We show how the electron-ion interaction potential (EIIP) amino-acid scale used in the ISM combined with a robust, high performance hydrophobicity scale can provide new insights for understanding protein structure/function and protein-protein interactions.

## Background

The problem of understanding protein structure, function and binding epitopes from the sequence remains challenging. In this paper, we extended the ISM, [1, 2] that combines an Electron Ion Interaction Potential (EIIP) amino acid scale, with a high performance hydrophobicity scale along with a novel DFT algorithm [3]. We show that ISM extended with other Digital Signal processing (DSP) approaches provides a unique way to understand the formation of protein domains and relation of primary sequence to the formation of secondary structure and the location of epitopes. We have used this method to understand virus-induced autoimmune diseases.

Lymphoproliferative disorders usually accompany persistent hepatitis C virus (HCV) infection [4]. This leads to a number of extra-hepatic manifestations [5] one of them being the Sjögren’s syndrome (SS). This syndrome, characterized by the kerato-conjuctivitis sicca and xerostomia, was proven to be associated with the presence of autoantibodies for the muscarinic receptor type 3 (M3R). The association was confirmed both in the primary and secondary forms of this syndrome [6–8].

Anti-muscarinic antibodies have been found to be crucial for exocrine gland dysfunction [6], but also for other accompanying autonomic dysfunctions (sudomotor, cardio-vagal and adrenergic functions) [9]. This important pathophysiological overlap between SS and HCV is further supported by data on cardio-vagal dysfunction in HCV [10–12]. Cardio-vagal dysfunction [13–18] is identified as one of the main reasons for increased mortality in chronic liver disease [19].

The mechanism of cardio-vagal lesion in HCV is still unknown. Some hypotheses have been proposed, like an immune mediated, common pathophysiological mechanism of hepatocellular damage and cardiac autonomic dysfunction [11, 12].

The acetylcholine muscarinic type 3 receptor (M3R), a G protein coupled receptor (GPCR), participates in the regulation of the heart rate and cardiac repolarization in animals [20] and humans [18, 21, 22]. Molecular signaling pathways participating in cardiac parasympathetic regulation, which include M3R, are still elusive, although some possible mechanisms have been proposed [23].

In addition to the heart, M3Rs are also present on progenitor hepatic cells [24]. This pool of cells is of particular importance for the regenerative process in diseased livers, including chronic HCV infection. Vagal stimulation, mediated through M3R, is therefore of crucial importance by increasing the pool of hepatocyte progenitor cells [24].

Since the M3R participates both in the regulation of the heart rate [20, 22], and hepatocyte turnover [23, 24], we hypothesize that the part of the possible common mechanism of the lesion of cardiac parasympathetic regulation and hepatocyte compartment [11, 12] is through immune-modulatory autoantibodies to M3R.

A number of studies have investigated the potential role of molecular mimicry in autoimmune disorders associated with HCV infection [25–27]. It is interesting to note that immunological cross-reactivity between proteins has been shown to correspond to a common frequency component in their informational spectra (IS) [28–31]. Our objective in this work was therefore to look for a common frequency in the IS (Information Spectrum) of M3R and the HCV proteins allowing us to provide strong evidence that the cross-reactivity is indeed due to *molecular mimicry*. We performed an ISM analysis of all proteins from the EUHCV database. Our analysis showed that the NS5A protein, particularly from HCV viruses genotype 1b, represents the most probable antigen which is cross-reactive with M3R. We also mapped a domain and binding epitope of NS5A representing a potential prognostic and therapeutic target for the cardiac dysfunction caused by the HCV virus.

## Methods

### Datasets

The dataset was created by extracting only the complete—full length HCV protein sequences from the GenBank polyprotein sequences. Human muscarinic acetylcholine receptor M3 was retrieved from UniProtKB/Swiss-Prot with accession number P20309. Prototype HCV protein sequences were retrieved from the EUHCV database [32].

### Informational spectrum method (ISM)

The ISM starts by assigning a numerical value to each amino acid in the sequence that best represents the physico-chemical property of the amino acid involved in the structure and biological activity of the protein. In this paper, we have used two different, but related methods to assign numerical values to each of the amino acids in the primary sequence—one is the electron-ion interaction potential (EIIP) [33–38], and the other is from a hydrophobic proclivity scale [3]. The next step in the ISM is to apply a Discrete Fourier Transform (DFT) and transform the protein primary (“time signal”) sequence into the frequency domain. In analyzing protein sequences, the relevant information is presented as an energy density spectrum (review in Reference 39) from the square of the Fourier Transform derived frequency amplitude coefficients obtained from the numerically encoded amino-acid sequence. The information defined by the sequence of amino acids is then available as an Information Spectrum (IS) representing a series of frequencies and their amplitudes. The total number of frequencies represented by the DFT amplitude/phase coefficients is limited by the Nyquist sampling theorem from information theory and the mathematics of the DFT algorithm. Each amino acid in the primary sequence is “sampled” at some “equal” frequency corresponding to the “time” intervals of one inter-amino acid step in the primary sequence. Due to the time and frequency symmetry, the reciprocal of each frequency f(n) from the DFT corresponds to a correlation of some step distance d(n) = 1/f(n) between amino acids in units of one amino-acid step distance. Per the Nyquist theorem, the maximum frequency (f(*n =* N)) will always be 0.5 corresponding to resolving a minimum inter-amino acid correlation distance of 2.0 amino-acids.

The IS frequencies f(n) corresponds to the distribution of structural motifs with defined physico-chemical characteristics responsible for the biological function of a protein and its binding affinities. Similarly, the amino-acid correlation distances d(n) provide information about the secondary and tertiary structure of the sampled protein, and about binding epitopes within proteins associated with long range inter-molecular attraction and binding. When comparing proteins which share the same biological or biochemical function, the ISM technique allows detection of protein-protein correlation pairs, whose common frequency amplitudes f(n) are specific for their common biological properties, or which correlate with their specific interaction. These common, dominant informational characteristic frequencies of the protein sequences are determined/pin-pointed by a Cross-spectrum or Consensus Informational Spectrum (CIS) (i.e. the Fourier transform of the auto-correlation function for the spectrum). In this way, any spectral component (frequency) not prominent in all compared IS spectrum’s are diminished and thereby eliminated. If one calculates a CIS (by multiplication of all the power spectrum values together at each corresponding frequency for each protein power spectrum) for a group of proteins having different primary structures, and finds strictly defined peak frequencies, it means that the analyzed proteins participate in mutual interactions or have a common biological functions.

We show in this paper how the ISM algorithm combines the method’s native EIIP scale with a robust hydrophobicity scale to provide a unique understanding into protein domains, functions and binding epitopes.

### The hydrophobicity scale

Hydrophobicity is a primary physico-chemical property of amino-acids associated with the structure and function of proteins. Hydrophobicity is a real and measurable force between aqueous clathrate membranes that spontaneously form about hydrophobic surfaces; a force which can operate up to 100-200nm [39–41]. Since there is a range of amino-acid physico-chemical properties with respect to each other and with respect to water, a hydrophobicity scale must reflect continuum differences ranging between extremes of molecular size, mass, geometry, polarity/hydrophilicity and molecular non-polarity/hydrophobicity. Amino-acid hydrophobicity scales (with various drawbacks/defects) have proliferated in the literature, but effectively they measure the contrast of the strength of interaction between amino-acids and water as a means of devising an amino-acid scale/index. The hydrophobicity scale used in this study has been developed to overcome current literature amino-acid hydrophobicity scale limitations in order to more accurately reflect the central, net resultant of amino-acid physico-chemical properties which result in amino-acid hydrophobicity’s (e.g. their interaction potentials with water) [3].

### The EIIP scale

The EIIP scale reflects the average energy of atomic valence shells and the resulting molecular orbitals of each amino-acid, acting as discrete electronic units, building up the electronic properties of peptides and proteins. The EIIP scale therefore reflects molecular properties from delocalized electron density. It is this latter property that allows proteins to have motifs with distinct wavelengths that support functions essential to specific protein functions and long range protein attractions [1, 2].

The joint dependencies (i.e. in phase relationships) of amino-acid EIIP and hydrophobicity properties reflect important functional epitopes or structural motifs within proteins, as we show in this current study. We postulate that the EIIP scale reflects the proclivity of a protein to form a QM/EM dipole capable of long range (up to 200 nm) attractions between proteins or proteins-substrates. Furthermore, we hypothesize that the inter-molecular QM/EM dipole interaction is transduced by aqueous clathrate shells (reflected by the hydrophobicity scale) at appropriate locations and transmitted through the bulk waters as a polaron mediated by aqueous phonons that operate in the low Terahertz frequency region.

### The ISM algorithmic steps for generating an informational spectrum analysis

1. Convert a protein sequence into its numerical sequences with EIIP and hydrophobicity index values
2. Apply the discrete Fourier transformation to the resulting two numerical series to generate the power density spectrum.
3. Apply the Cross Informational Spectrum algorithm (i.e. multiply the corresponding frequency amplitudes squared) to the set of DFT spectral energies and obtain the CIS consensus spectrum to recover the dominant relational/inter-correlation frequency/frequencies.
4. Perform the sliding window DFT algorithm calculating signal to noise (S/N) calculations for each window location for each of the dominant CIS frequencies to identify the hot spot location(s) for each protein corresponding to binding epitopes/active sites being indicated by the ISM method.
5. For the hybrid algorithm introduced in this paper, for each protein run the Cross-Correlatoin Dependancy (CCD) analysis on the product of the EIIP encoded sequence and the hydrophobicity encoded sequence in order to recover the primary binding epitopes and confirm the conventional ISM hot spot locations.

### Core equations describing the discrete fourier transform (DFT)

The protein primary sequence, which has been numerically encoded into a number sequence representing the alternate numerical description of a protein, is processed with the Discrete Fourier Transformation (DFT) defined according to the following equations:

1 $$ X(n)={\displaystyle \sum_{m=1}^Nx(m){e}^{-i2\pi nm/N}},\kern0.48em n=1,2,\dots, N/2 $$

where m is the summation index, x(m) is the m^th^ member of a given numerical “signal” series (from a transformed, encoded primary protein sequence in our case), N is the total number of points in this series (in our case the number of amino-acids), n is the number of a discrete frequency (ranging from 1 on up to N/2) in the DFT, X(n) are the discrete Fourier transformation amplitude coefficients corresponding to each discrete frequency n, and 2π*(n/N) is the phase angle at each given m in the amino-acid series of the protein in question. These X(n) amplitude coefficients and corresponding Y(n) phase coefficients describe the amplitude, phase and frequency of sinusoids, which are a frequency based decomposition of the signal, that both describe the original protein signal and can be used to reconstitute/recover the original signal. The complex discrete Fourier transformation defines both the amplitude spectrum and the phase spectrum, where the complete information about the original sequence is contained between both spectral functions. However, in the case of protein analysis, the relevant information is primarily presented in energy density spectrum (for review see [42]), which is defined as follows:

2 $$ S(n)=X(n){X}^{*}(n)=\left|X(n)\right|{}^2,\kern0.36em n=1,2,\dots, N/2 $$

### Cross-codependency (EIIP, hydrophobicity) DFT analysis

A modified version of the ISM DFT algorithm using the Euler formula was also used on a derivative metric calculated by multiplying each given protein amino-acid hydrophobicity times its corresponding EIIP value. This secondary metric will reflect the joint biophysical effects of amino-acid molecular orbital electrons (from atomic valence shell electrons), amino-acid polarity/polarizability and amino-acid contact interactions with aqueous shells (when the amino-acids are exposed to water). The amino-acid Hydrophobicity values are given in Table 1.

**Table 1:** The Hydrophobicity proclivity values used in hybrid algorithm to encode the amino acids

Amino acid	Hydrophobicity
Phe	0.0688
Leu	0.0579
Ile	0.0349
Met	0.2213
Val	0.1427
Pro	0.7123
Thr	0.6599
Ser	0.7074
Ala	0.4925
Tyr	0.4523
His	0.6763
Gln	0.8692
Asn	0.8350
Lys	0.9651
Asp	0.9157
Glu	0.8974
Cys	0.2650
Trp	0.3403
Arg	0.9091
Gly	0.6582

A Cross-CoDependancy (CCD) analysis of amino-acid electronic and hydrophobicity in proteins detects whether or not the primary sequence of a given protein will have dominant and characteristic ISM frequencies for EIIP encoding and Hydrophobicity encoding that will have intersecting sequence “hot spots” that define important long range QM/EM dipole attraction sites and/or binding epitopes. If codependent hot spots exist in a given protein, then the periodic physico-chemical properties reflected by the EIIP and Hydrophobicity indices are basically in “phase.” The protein sequence hot spots are recovered by the standard positional ISM Signal to Noise ratio (S/N) analysis as illustrated in the results section of this paper [43].

The CCD-DFT analysis utilizes the Euler formula to strip out the frequency amplitudes:

3 $$ {e}^{i\theta }= \cos \left(\theta \right)+i \sin \left(\theta \right) $$

The Euler formula is at the heart of the DFT method, where an exponential function is related to the sum of two sinusoidal functions. In this decomposition relationship, cos(Ɵ) represents the amplitude and sin(Ɵ) reflects the phase of the “time” varying “signal.” Here the phase angle theta (Ɵ) is equal to 2π*(n/N) at each sliding window location m where these variables have the same definition as the equation (1) variables. The CCD-DFT will only utilize the amplitude portion of the protein primary sequence signal as reflected by:

4 $$ Dc\left(m,n\right)= EIIP\ast Hydrophobicity\ast \cos \left(\theta \right) $$

Similar to the way the ISM S/N analysis proceeds, the CCD-DFT analysis computes successive CCD-DFT’s for sliding fixed width windows of the secondary scale. The amplitudes of the CCD-DFT windows are calculated using:

5 $$ Fd(n)={\displaystyle \sum_{m=1}^{Ww}Dc\left(m,n\right)} $$

Where Ww is the CCD-DFT window width that is calculated by selecting the largest of the EIIP or Hydrophobicity dominant correlation distances, as calculated from the reciprocal of the respective dominant ISM frequencies, which is rounded to the nearest integer. We show later, the Ww calculated for this ISM analysis of the HCV NS5A protein is 6. The ratio of Ww to the other correlation distance is expected to result in a ratio value near an integer (to which it will be rounded), if the phase relationship between the EIIP signal and the Hydrophobicity signals are close and one of the signals is close to a sub-harmonic of the other.

Microsoft Office 2010 Excel was used to calculate and plot the Fd(*n =* 0) and Fd(*n =* 1) series, where each Fd() is calculated for the sliding window of width Ww. The number of Fd() values is N - Ww. The Excel trend plot feature was used to plot the Fd() values. The Excel trend plot feature does a smooth curve fit of calculated values, which is related to a second order b-spline smoothing method. The Fd (*n =* 1) points result in a sinusoid of wavelength of 6 amino-acid steps. The Fd(*n =* 0) represents a special case where cos(Ɵ) = 1 and Fd(*n =* 0) is simply the sum of the Dc = EIIP*Hydrophobicity values for each sliding window of size Ww. The Fd(*n =* 0) coefficients represent the outer amplitude envelope of all sinusoid frequencies within each sliding window. The Fd(*n =* 1) plot sinusoid peaks shows the location of each putative intersection (phase crossing) of the EIIP and Hydrophobicity metrics (amino-acid sequence physico-chemical properties), where we also see the Fd(*n =* 1) positive peaks touch the Fd(*n =* 0) amplitude envelope. Additional confirmatory work was conducted using standard FFT methods on the NS5A domain I amino acids for both power (amplitude squared) and phase coefficients squared [2].

### Docking

Docking simulations were done on a HEX Server [44]. For protein-protein docking, using results from the ISM, interface residues were set from #513 to 551 in Muscarine M3 receptor (structure PDBID 4DAJ) and from #171 to 187 in HCV NS5A protein domain (structure PDBID 3FQQ). The range angles, which determine conformational space of both proteins, with origins in centroids of residue selections, were set to 45°. The number of output solutions was set to 100. The solution with best docking score was selected for further analysis.

## Results

Calculation of the cross-spectrum (CS) between M3R and each protein from the EUHCV database revealed that maximal amplitudes and signal-to-noise ratio (S/N) corresponds to the CS relationship between the M3R and HCV NS5A proteins, and that f(0.158) represents the common frequency component in IS for these two proteins using the EIIP scale.

**Fig. 1:**
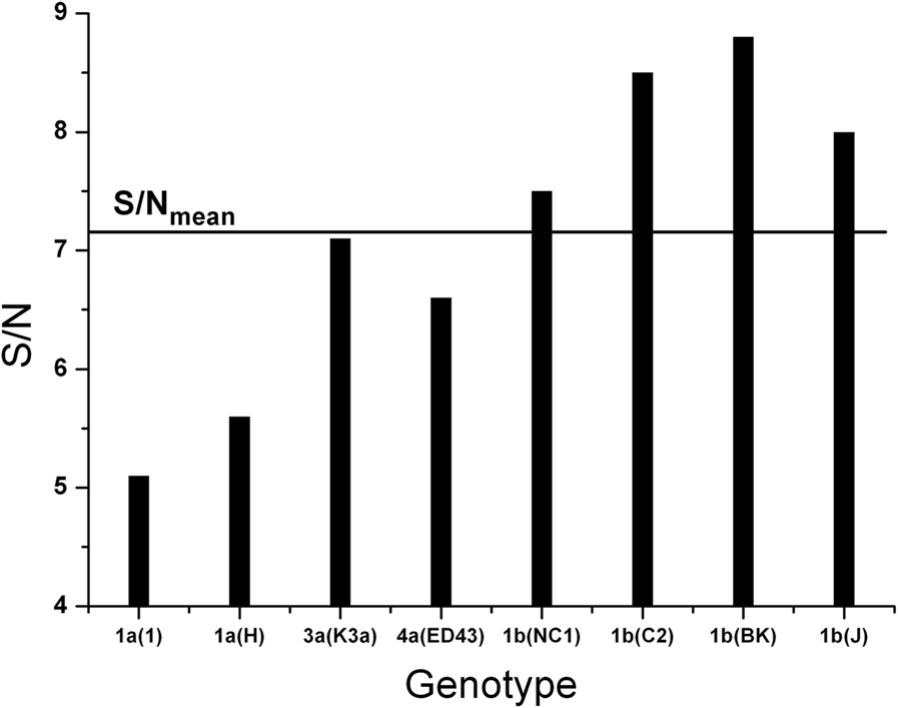
S/N values from the cross-spectra(CS) of M3R and prototype NS5A HCV sequences from EUHCV database

Because of the variability of the NS5A protein, we further investigated the IS cross correlation of both the M3R protein and the NS5A protein from prototype isolates of different HCV genotypes. As can be seen in results presented in Fig. 1, the highest S/N values correspond to HCV genotype 1b.

**Fig. 2:**
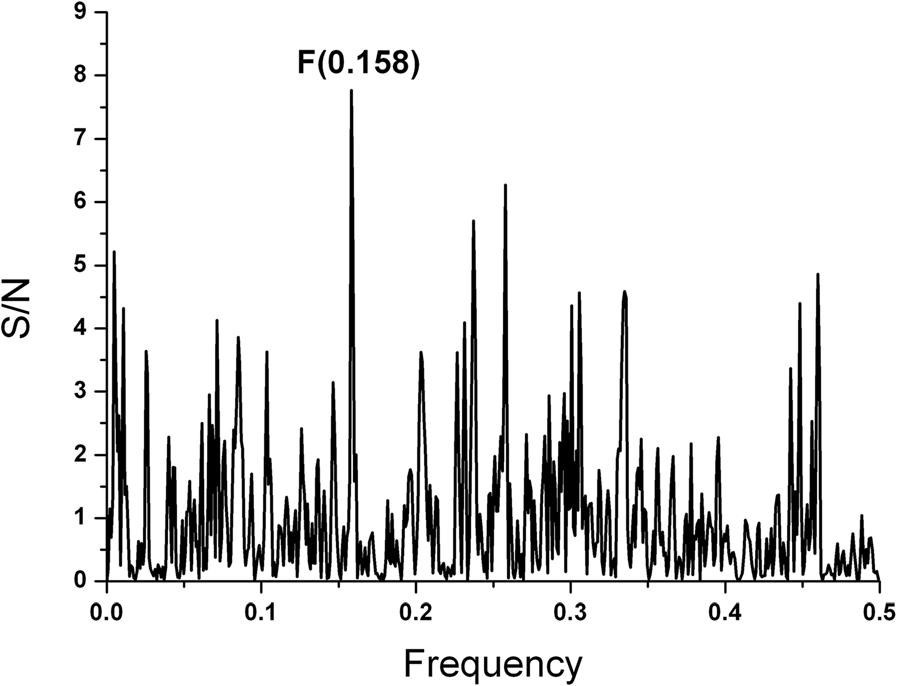
Cross-spectrum (CS) of M3R and NS5A(BK)

**Fig. 3:**
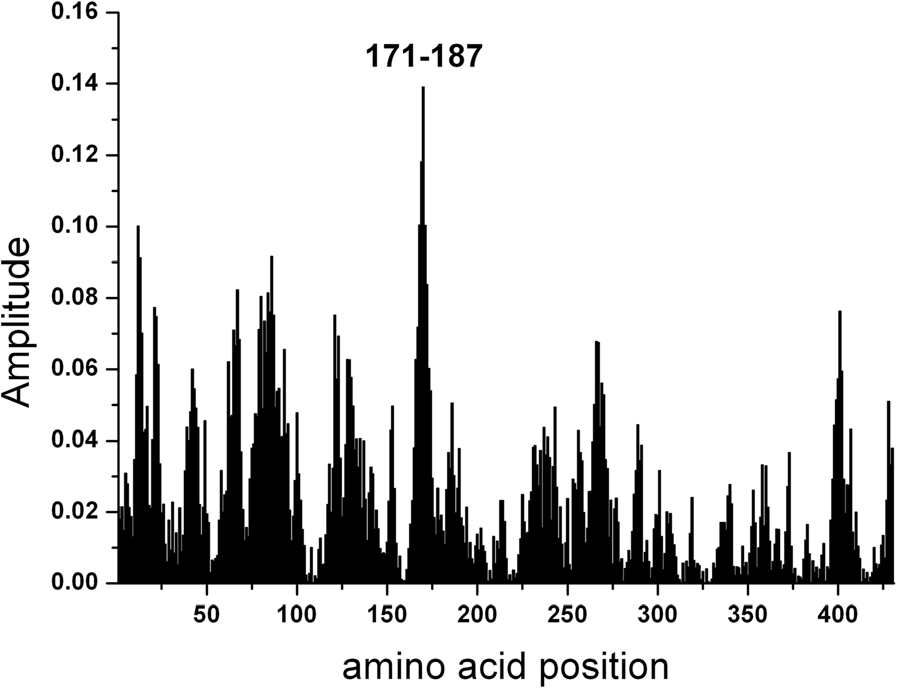
Mapping of the domains with maximal contribution to the frequency component F(0.158) in the informational spectrum of NS5A(BK)

To identify the epitope of NS5A which would be involved in immunological cross-reactivity, the EIIP based ISM analysis of this protein from the prototype HCV BK isolate was performed. The dominant peak in CS of M3R and NS5A(BK) corresponds to the frequency f(0.158) (Fig. 2). Computer-assisted screening of NS5A(BK) with peptides of different lengths revealed that domain encompassing residues 171–187, gives an essential contribution to the information corresponding to the frequency f(0.158) (Fig. 3). According to the previously reported results of similar analysis [28–31], it could be concluded that this domain of NS5A(BK) is responsible for its immunological cross-reactivity with M3R.

**Fig. 4:**
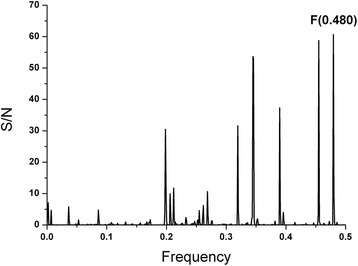
Cross-spectrum (CS) of M3R and NS5A(BK) (Hydrophobicity scale)

In order to predict accessibility of the potential signal epitope at AA 171–187, we performed an ISM analysis of NS5A(BK) using a high performance, high fidelity Hydrophobicity scale [3] as a molecular descriptor of amino acids instead of the EIIP scale (Fig. 4). The dominant peak in this CS corresponds to the frequency f(0.480), which corresponds to a 1/f correlation distance of 2.08 AA. There is a secondary peak at f(0.460) with a S/N amplitude near that of the primary peak, having a 1/f correlation distance of 2.2 AA. Since the Beta strand/sheet periodicity is right at 2.2 AA, these two peaks suggest that the NS5A Domain I has secondary structure that is dominant in Beta sheets, as the Domain I X-Ray crystal structure confirms (Fig. 7). The EIIP dominant peak f(0.158) has a 1/f correlation distance of 6.33 AA. The ratio of the EIIP to the hydrophobicity scale correlation distances yields a ratio of 3, which indicates that the EIIP scale dominant frequency in Domain I is a sub-harmonic of the Hydrophobicity dominant frequency. This relationship implies a pattern essential to the Domain I structure and function.

The supplementary FFT analysis (length of 256) of the encoded domain I amino acids using the EIIP, hydrophobicity and joint (multiplied) scales generally confirms these primary functional wavelengths. Interestingly, the squared phase coefficient spectrum of the joint scale had two primary wavelengths of 2.15 as opposed to 2.08 and 6.24 as opposed to 6.33. These latter wavelength results are essentially the same as the native ISM method findings within the wavelength resolutions imposed by these basic DSP methods.

**Fig. 5:**
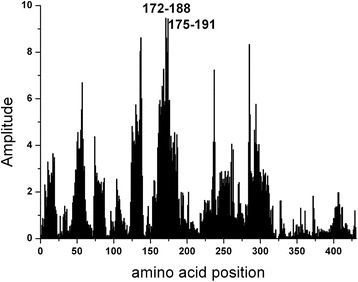
Mapping of the domains with maximal contribution to the frequency component F(0.480) in the informational spectrum of NS5A(BK) (Hydrophobicity scale)

The computer-assisted scanning of NS5A(BK) showed that overlapping domains 172–188 and 175–191 give the biggest contribution to the dominant frequency f(0.480) in the IS based on the Hydrophobicity scale [3] values of amino acids (Fig. 5), which overlap the primary epitope indicated by the EIIP ISM results. These two bands also correspond to two signal epitope patterns (valleys dipping below 0.15) revealed in the CCD-DFT analysis (Fig. 7) with a primary minima at AA 174 and secondary minima at AA 187, respectively.

**Fig. 6:**
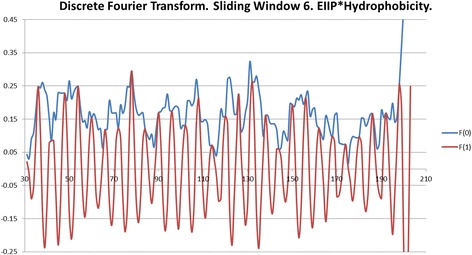
NS5A Domain I CCD-DFT showing the major signaling epitope near AA #175

It is important to note that the locations of the primary signal epitope, obtained by both the EIIP and hydrophobicity [3] scales/descriptors, significantly overlap (Fig. 6).

**Fig. 7:**
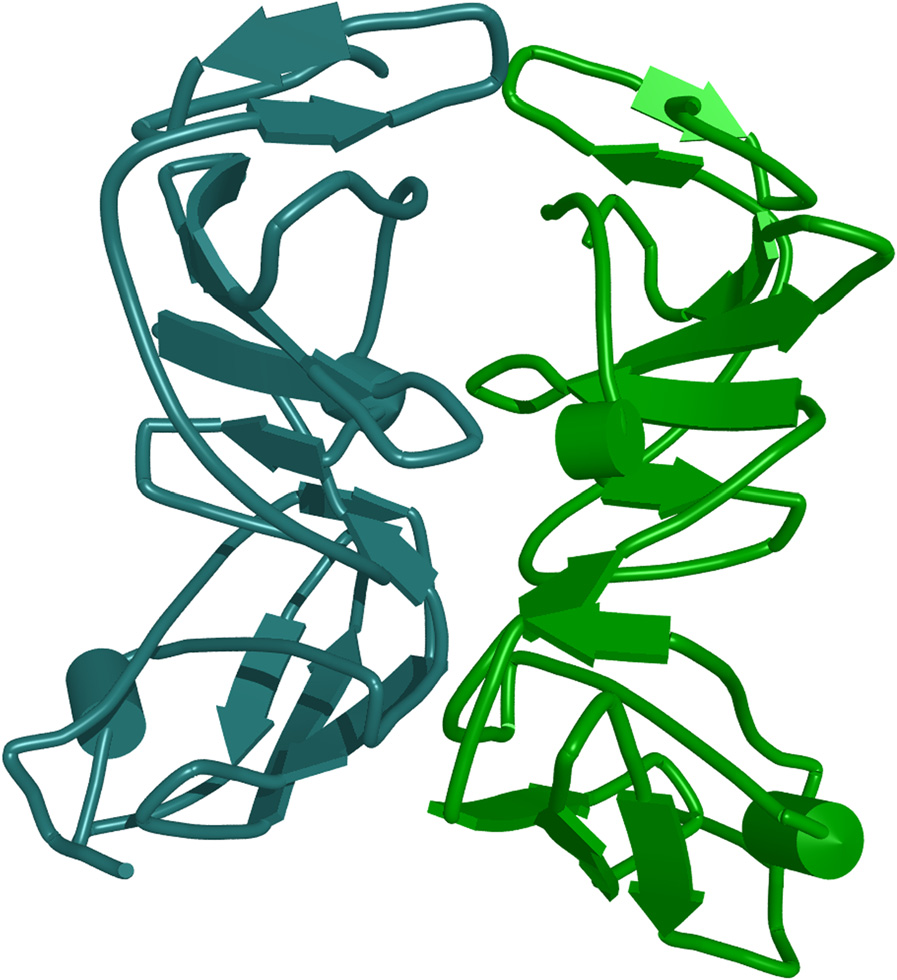
Ribbion diagram of PDBe 3fqq, which is the crystal structure of Domain I of the NS5A protein

The plot (Fig. 6) represents a Discrete Fourier Transform (DFT) analysis (6 amino-acid sliding window) of the HCV NS5A protein Domain I, using a convolving (sliding multiplication) of the EIIP value and the Hydrophobicity value for each amino-acid selected (31–207) from Domain I. A sliding window of 6 AA was chosen for the analysis owing to the fact that the EIIP dominant IS 1/f correlation distance is 6.3 AA. The amino-acids selected from Domain I are those from the Domain I X-Ray crystal structure in the PDB (3fqq, Fig. 7), which is the only portion of the HCV NS5A protein with a solved structure in the PDB. The EIIP and Hydrophobicity product forces a synchronization between the two metrics for the CCD-DFT analysis, but it also reflects important structural and functional relationships within the NS5A Domain I amino acids.

In particular, the pronounced valley’s in the blue Fd(0) trace dipping below 0.15 represent important regions of phase synchronization between the electronic (EIIP) configuration and the residue hydrophobicity’s, which reflect solvent like residue-residue contacts (senso-lato) and/or important residue contacts with the solvent (water, senso-stricto). The lowest hydrophobicity values represent the most hydrophobic residues, which then range up to higher values near one representing polar and then ionic residues. Hydrophobicity in the sense of the Hydrophobicity scale means that the hydrophobic residue contact with water promotes water-water surface film/membrane (clathrate) interactions. The Hydrophobicity scale also reflects information from the mass, volume, surface area, secondary group geometry and entropy of the 20 natural amino acids [3].

Further insight into the plot above may be gleaned from Table 2 below, with notes reflecting both background information and specific observations from the CCD-DFT plot, which nails the primary epitope pattern of the NS5A protein domain #1 at around residue 174 (or 171–182 from the valley width), which is consistent with the primary ISM result. There may be as many as 6–8 signaling/binding epitopes within Domain I of the NS5A protein, with significant size Fd(0) valleys, dipping below 0.15, in the Fig. 6 plot. This pattern is also seen in the similar number of peaks shown in the amplitude plots (Figs. 3 and 5). The CCD-DFT analysis not only confirms the ISM result, but adds further insight into the structural and functional features of the NS5A protein domain I. A description of the location and effective range of the Fd(0) values in the Fig. 6 plot is in the Table 2 below.

**Table 2:** A list of the NS5A domain I epitopes indicated by the CCD-DFT analysis

Fd(0) valley (AA#) minima	Putative width of epitope effect range (#AA) about the epitope valley minima
32	(end of N terminal membrane anchor motif)
42	10
66	24
90	24
115	25
144	29
175	31
187	12

The NS5A domain #1 crystal structure (Fig. 7, PDBe 3fqq) shows the secondary structure is almost all beta sheets. The valleys in Fd(0) represent distinct and functionally active correlations between EIIP and the Hydrophobicity. Position #175 represents the epicenter of the domain #1 primary epitope, as this is the minimum Fd(0) amplitude valley. Position #198 on represents a radical shift in the amino-acid pattern signaling the end of domain #1. Positions 174–178 are a Beta strand (TFLV) and positions 180–183 are a Beta strand. These 2 strands are part of a 3 strand Beta sheet with a small intercalated alpha helix. The bound Zinc (Zn + 2) ion is located right at the alpha helix in the primary epitope area. The putative epitopes reported in Table 2 are consistent with reported literature epitopes within NS5A, and in particular the primary NS5A epitope has been reported as a NS5A dimer binding site (a 2 x 3 = 6 strand beta sheet), where the dimer is important for viral replication [45, 46]. In silico docking studies and site directed mutagenesis binding studies with a known NS5A activity inhibitor implicate sensitive AA sites: 21,24,28,30,31,38,54,56,58,62,75,92,93; where AA# 21&24 are in a membrane anchor alpha helix and the balance of these AA sites are within epitope ranges indicated in Table 2 [47].

The primary Domain I signal epitope is located in the center left of the Fig. 7, which is a motif consisting of a three strand Beta sheet and a small Alpha helix, and the primary signal epitope is located on two of the Beta strands in the indicated motif.

**Fig. 8:**
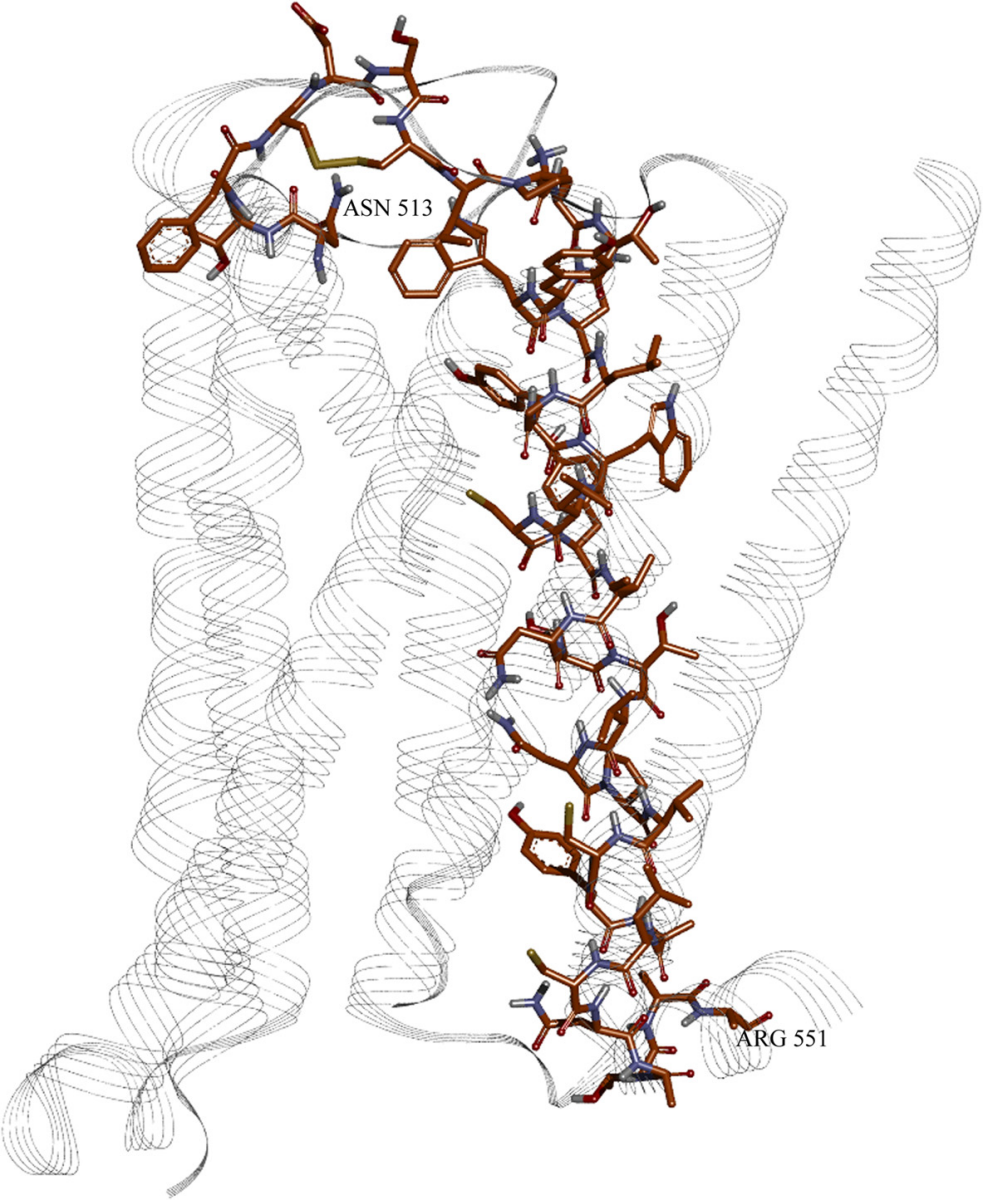
The muscarinic M3 receptor (3FQQ) with marked 513–551 region

The reported domains of the muscarinic M3 receptor and NS5A that showed compatibility based on the hydrophobicity spectrums are presented on Fig. 8.

**Fig. 9:**
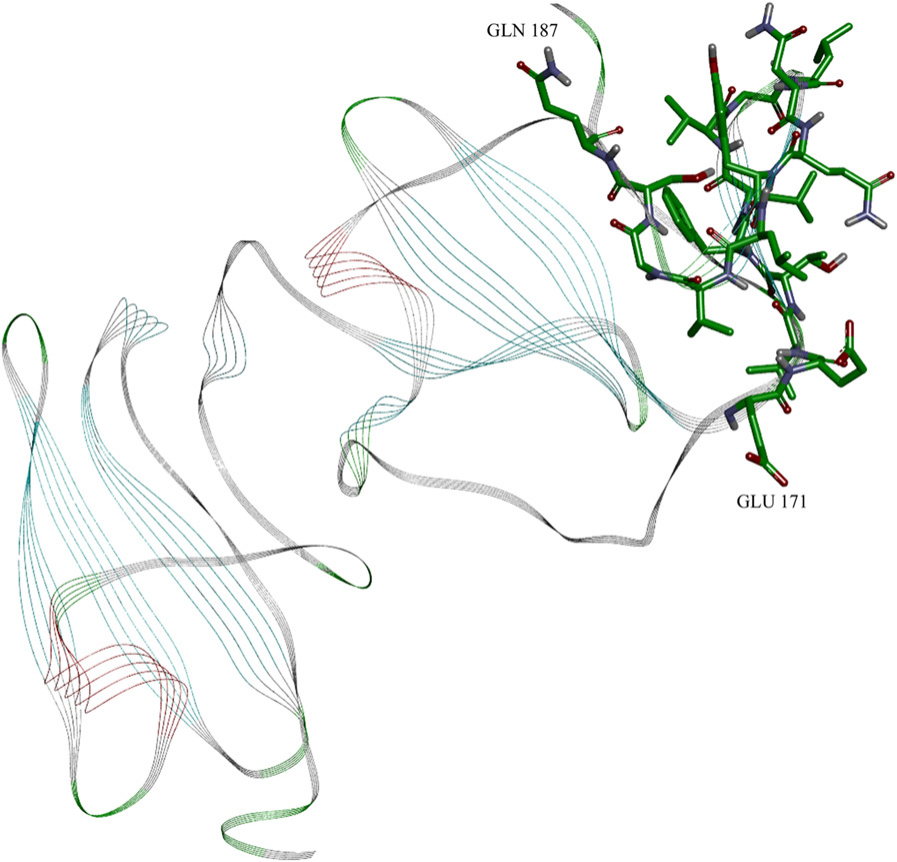
The NS5A protein with marked 171–187 region, with amino-acids drawn in green stick

**Fig. 10:**
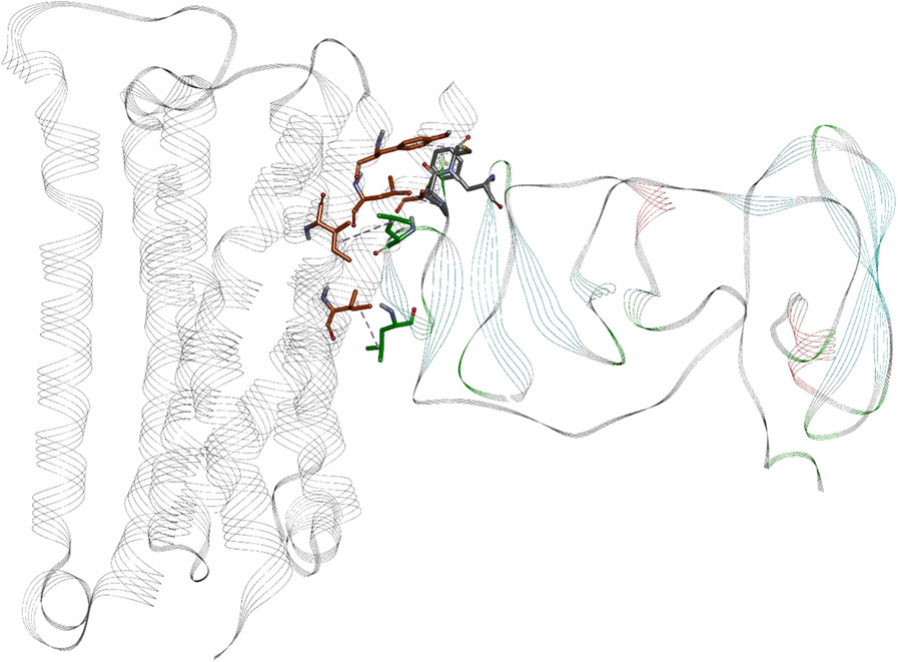
The docked conformation of M3 and NS5A, with established hydrophobic contacts

**Fig. 11:**
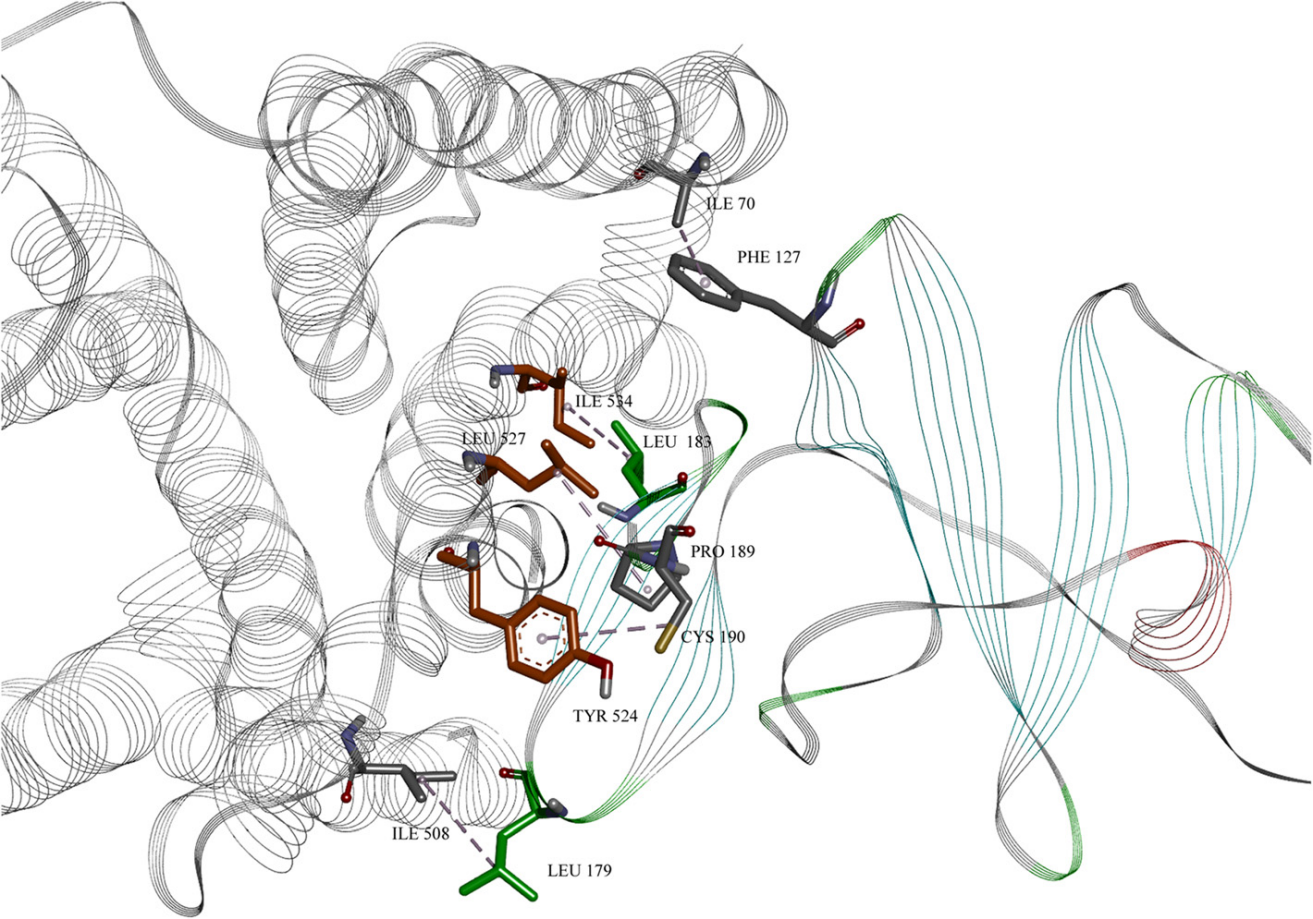
Detailed established hydrophobic interactions between M3 and NS5A. Amino-acid residues of proposed region in M3 are colored in orange; in NS5A are in green. The rest are colored in grey, although not in proposed regions, but with established hydrophobic interactions

The domains are drawn in stick and colored in orange and green, respectively. We have also performed rigid protein – protein docking, targeting proposed domains, using pdb structures 3FQQ [48] and 4DAJ [49]. The docking results (Figs. 8, 9, 10 and 11) gave a compatible solution (among the best ten confirmations), showing established hydrophobic interactions between domains of two proteins.

The interactions are presented in Fig. 8 and quantified in Table 3. Although the indicated interactions are not completely within the primary predicted region of the primary epitope, the established hydrophobic interactions are enough close to validate our assumption possibility of interaction between HCV NS5A with M3R. Moreover, as we compare the contacts in Table 3 with the epitopes noted in Table 2, we see that all of the contacts recovered in the contact analysis derive from amino-acids within either the primary epitope or within two secondary epitopes of the NS5A domain I.

**Table 3:** Hydrophobic contacts between muscarinic M3 receptor and NS5A, with corresponding amino-acid residues, highlighted atoms and distances and angles between them

Muscarinic M3 receptor (atom)	NS5A (atom)	Distance/Å	Bond angles/degrees (atoms)
ILE 70 (CB)	PHE 127 (CG-CD1-CD2-CE1-CE2-CZ centroid)	3.668	31.747 (ILE70 (CB)—PHE127 (centroid)—PHE 127 (CD1))
TYR 524 (CG-CD1-CD2-CE1-CE2-CZ centroid)	CYS 190 (SG)	4.601	21.30 (TYR 254 CZ-TYR 254 centroid—CYS 190(SG))
LEU 527 (CG)	PRO 189 (CB-CG-CD centroid)	5.244	133.20 (LEU 527(CG)—PRO 189(CA)—PRO 189(centroid)
ILE 534 (CG)	LEU 183 (CG)	4.326	48.88 ILE 534 (CG)—LEU 183 (CD2)—LEU 183(CG)
ILE 508 (CG)	LEU 179 (CG)	4.994	72.54 ILE 508 (CG)—LEU 179 (CD2)—LEU 179 (CG)

Finally, to determine domain which gives essential contribution to the information represented by the frequency F(0.158) in MR3, the ISM computer-assisted screening of the MR3 primary structure was performed. Identified domain denoted VIN_M3R_ is within the C-terminus of MR3, encompassing the third extracellular loop (ECL3) and the TM alpha helix #7 (residues 517–551). According to the ISM concept, this region would be immunologically crossreactive with NS5 or could be involved in the direct M3R/NS5A interaction.

## Discussion

### DSP applications of the EIIP and hydrophobicity scales & experimental support

The ISM has been successfully applied in structure-function analysis of many different protein and DNA sequences, prediction of biological function of novel proteins, de novo design of biologically active peptides, assessment of biological effects of mutations, and identification of new therapeutic targets [36, 42].

For example a ISM DSP analysis using the EIIP scale could find known binding sites (hot spots) for 5 chemotherapeutic Tubulin binding agents and could also determine relative differences in binding affinities to these hot spots as applied to 10 human tubulin isotypes [50].

Similarly, a FFT power spectrum analysis of anti-microbial peptides found distinct and matching peaks in power spectra using hydrophobicity as the primary scale (Kyte & Doolittle hydropathy index) and confirmed with a composite scale with 4 other AA metrics [51].

An ISM like DFT method has been published [2] where hydrophobicity and EIIP were found to be especially robust AA physico-chemical scales (with 611 physico-chemical scales evaluated) for analyzing Influenza A strain proteins with identification of characteristic frequencies as found with the ISM DSP methods in this study. The ISM like DFT methods showed that the phase (sin()) coefficients squared spectrum was generally as informative for finding characteristic frequencies as was the power spectrum [2].

A DSP method has also been developed [1, 52] using a different algorithm than DFT derived methods, such as the ISM, using primary protein sequences encoded with a simple modified Nozaki–Tanford–Zimmerman hydrophobicity scale. This DSP method attempted to find “autocorrelation waves” or “hydrophobic eigenmodes,” using a lagged auto-covariance matrix decomposition and all poles power spectral and wavelet transformation. In practice this method finds characteristic structural/functional frequencies similar to the ISM method. This method was applied to Type I Tyrosine Kinase-coupled receptors and GPCR receptor proteins giving primary (often structural, such as in GPCR’s 7 transmembrane alpha helices) and secondary hydrophobicity activity wavelengths, that were then used to efficiently design (30 %–80 % hit rate) receptor agonist/antagonist binding peptides (aptamers, 8–20 residues) against these receptors (external loops), possessing matching inverted hydrophobicity structural modes. These results were confirmed in vivo, both at cellular and organismal levels (rat).

This same lagged eigen-function method was used to design aptamers against a globular protein (beta-galactosidase) and verified with temperature dependent kinetic studies with an ELISA method and the with Van't Hoff relation. The results indicated a classical linear free energy (enthalpy, entropy) relation characteristic of hydrophobic interactions of small and large bio-molecules in water. There was an entropy–enthalpy compensation relationship which showed signs of both competitive and non-competitive interactions [1]*.*

Some of the references to studies using DSP methods to analyze protein primary structure using hydrophobicity scales and the EIIP scale illustrate the power of analyzing protein structure and function with these DSP methods, such as the ISM platform, are far more than mathematical curiosities’, but rather have experimentally supported real world applications.

### Implications for clinical research

The pathophysiological overlapping between SS and HCV, presence of anti-M3R antibodies in SS, the role that M3R plays in the regulation of the heart rate and cardiac repolarization [18, 20–22], has led to the assumption that cardiovagal dysfunction in HCV patients is caused by anti-M3R antibodies elicited by HCV proteins, or by their direct interaction with M3R. In order to identify the HCV protein which possibly is cross-reactive with M3R, or which binds to this receptor, we performed an ISM analysis of the HCV proteome. Our analysis has revealed that the NS5A protein represents the possible interactor with the M3R receptor, or that this viral protein elicits antibodies which modulate function of this receptor. Comparison of the ISM of NS5A from different HCV genotypes showed that genotype 1 has the highest value of S/N on the frequency f(0.158) suggesting that these proteins are the most probable HCV pathological modulators of the biological activity of M3R (Fig. 1). Computer-assisted screening revealed that domains VIN_NS5A_ (residues 171–187) and VIN_M3R_ (residues 517–551) are probably involved in immunological cross-reactivity or in direct interaction between NS5A and M3R. The VIN_M3R_ peptide corresponds to part of the extracellular loop 3 and the TM alpha helix #7.

Informational similarity could result in immunological cross-reactivity which was experimentally shown in previous studies [29]. In the current study we have identified AA 171–187 of the HCV NS5A as the domain contributing the most to the characteristic information important for cross-reactivity. Evidence from various studies carried out with NS5-derived antigens has demonstrated that they are not as immune-reactive as the other antigens [53, 54], and could be less specific [55]. In contrast to earlier findings, Rodríguez-López et al. [56], we have found the NS5A domain I region to be among the most immunogenic. In accordance with the present results, in their experiments studying the immunogenicity of the variable regions of HCV proteins by ELISA using synthetic peptides from 120 variable regions in sera from HCV-infected cells, they have found the primary epitope, mapped to region 2144–2149 (172–177 NS5A) inside the ISM identified NS5A domain I with the motif (2144) DVTFQV (2149). This finding provides further support for our hypothesis of NS5A as possible immunogenic epitope.

Another important data is that ISM identified domain VIN_M3R_ in M3R is overlapping with functional epitopes of M3R reported by Koo et al. [57] (residues 517–527) and Tsuboi et al. [58] (residues 517–530) within the M3 third extracellular loop which interacts with autoantibodies from SS patients. This finding suggest that it cannot be excluded that this antibodies could be also elicited against NS5A in SS patients infected with HCV, supporting the finding of an involvement of HCV in the development of SS in a specific subset of patients [59].

Data from the literature support a link between antibodies against M3R and functional inhibition of the M3R receptor. The agonist binding site occupation by antibodies against M3R was reported as the key mechanism for loss of M3R function [60] and new evidence on the role of antibodies against M3R in M3R internalization has been presented [61]. Also it should not be ignored that the TM alpha helix #7 has been reported [49] as an important allosteric binding site and consequentially a chance exists that an impaired function of M3R could be mediated through allosteric inhibition by antibodies against M3R.

Another possibility according to the ISM results is direct interaction between NS5A and M3R. Based on ISM and protein-protein docking results, we propose that, in conditions of a water medium, there is possibility that those two proteins interact and form a complex, like that of an antigen-antibody. The stabilization is based on hydrophobic interactions of targeted regions, which are favored in the water medium. NS5A can act as potential inhibitor of M3R, or could result in impaired trafficking of the M3R to the cell surface. In order to test all these hypothesis and potential molecular mechanisms of NS5A and M3 interaction, experimental studies can be designed with guidance from our DSP analysis. These experimental data should put novel light on the subject of progenitor hepatic cell apoptosis and the phenomena of HCV overlap with Sjöngren syndrome [5], or parasympathetic lesion [11].

Finally, the N-terminal of M3R region, which was identified as the region contributing the most to the characteristic f(0.158) frequency, could be the region of special interest for the pharmacological design of the substances that could block cross-reaction with M3R [30, 31], and in this way preventing cardiac dysautonomy and M3R dependent hepatocellular damage in HCV patients. It is also plausible that the mutations in this region or in other regions that cause the conformational changes of M3R could induce protective or susceptibility effects for different extra-hepatic manifestations of HCV caused by the lesion of M3R.

## Conclusions

In conclusion, our present study could have important clinical implications, such as the possibility of designing of aptamer peptides to sequester viral proteins in order to preclude cellular receptor binding and resulting in aptamer peptide-protein complexes that could be cleared by the immune system. Presented advances in the ISM platform could empower DSP methods to become mainstream tools for analyzing protein structure/function and to bring about a foundation for better therapeutics.
